# Effect of Syllable Articulation on Precision and Power Grip Performance

**DOI:** 10.1371/journal.pone.0053061

**Published:** 2013-01-09

**Authors:** Lari Vainio, Mirjam Schulman, Kaisa Tiippana, Martti Vainio

**Affiliations:** 1 Division of Cognitive and Neuropsychology, Institute of Behavioural Sciences, University of Helsinki, Helsinki, Finland; 2 Phonetics and Speech Synthesis Research Group, Institute of Behavioural Sciences, University of Helsinki, Helsinki, Finland; The University of Western Ontario, Canada

## Abstract

The present study was motivated by a theory, which proposes that speech includes articulatory gestures that are connected to particular hand actions. We hypothesized that certain articulatory gestures would be more associated with the precision grip than with the power grip, and vice versa. In the study, the participants pronounced a syllable and performed simultaneously a precision or power grip that was theorized to be either congruent or incongruent with the syllable. Relatively fast precision grip responses were associated with articulatory gestures in which the tip of the tongue contacted the alveolar ridge ([te]) or the aperture of the vocal tract remained small ([hi]), as well as gestures that required lip protrusion ([pu]). In contrast, relatively fast power grip responses were associated with gestures that were produced by moving the back of the tongue against the velum ([ke]) or in which the aperture of the vocal tract remained large ([hα]). In addition to demonstrating that certain articulatory gestures are systematically connected to different grip types, the study may shed some light on discussion concerning sound symbolism and evolution of speech.

## Introduction

An accumulating body of evidence supports the view that manual processes are intimately linked to language processes. For example, McNeill's [1] studies have suggested that manual gestures (i.e., communicative hand movements) are as integral part of language as are words. According to McNeill's and many others' view [2], speech and gesture form a unitary system. A related, increasingly popular view suggests that speech has evolved from manual gestures [3]–[6]. For example, the fact that the same left cerebral hemisphere plays a dominant role in the control of praxis and language [7]–[8] is in line with the gestural theory of language. The theory is also supported by brain imaging studies showing that activity in Broca's area, which is known to be linked to speech production, is also modulated by grasping and manipulation [9]–[10], observation of manual actions [11] as well as imitation [9] [12] and observation of gestures [13] [9].

Importantly for the purpose of the present paper, the system that is involved in grasping has been suggested to have a central role in the evolution of language. For example, Arbib [14] has proposed that protospeech has evolved from manual-based communication. According to this view, the evolution of grasping, and an imitation system for grasping, are the initial milestones on which the system that we nowadays use for speech has started to evolve. This view is in line with neurophysiological evidence showing that in monkeys, the same neurons are involved in commanding grasp motor acts with both the mouth and the hand in the premotor area F5, which is considered the homologue of human Broca's area [15]. This evidence has been proposed to reflect double grasp preparation processes of the distal effectors that are typically used to accomplish the grasp actions involved in ingestive behaviour [16]–[17]. Eating usually involves grasping a piece of food with the hand, bringing the food to the mouth and finally grasping it with the mouth (or taking possession of the food with the mouth). Although these neurons were most likely originally associated with eating behaviour, it has been proposed that they were later adapted for communicative purposes [18] [2] transferring the repertoire of manual grasps to articulatory gestures. Moreover, this notion of the double grasp neurons can be associated with a more general model of action planning, according to which the neuronal populations in premotor areas can be specialized in higher-order aspects of movement that can incorporate multiple effectors. For instance, electrical stimulation of neurons in this area can produce ethologically relevant behaviors such as closing the hand grip while bringing the hand to the mouth and opening the mouth or turning the head to one side and moving the arm up, as if to protect the face from a threatening impact [19].

Gentilucci and his colleagues have shown elegant behavioural evidence of the interplay between mouth movements and hand grasping. They have, for example, demonstrated that grasping large objects in comparison to small objects increases lip kinematics and voice spectra parameters (particularly the first formant) associated with pronunciation of syllables when the pronunciation occurs simultaneously with the grasp execution [20]. Similar effects were observed when the participants simply watched grasping of large or small objects [21]. More recently, Gentilucci and Campione [22] have shown that the interplay between the mouth and hand processes also operates in the other direction – from mouth to hand. When the participants held their mouth open, finger shaping associated with grasping was relatively large, whereas when the mouth was closed, finger shaping was smaller. A similar effect was observed when participants were asked to pronounce an open vowel [α] or a close vowel [i]. The pronunciation of the open vowel increased finger apertures of the precision grip in comparison to the close vowel.

### Potential associations between articulatory gestures and precision or power grips

The current study investigates whether some articulatory gestures are associated with the precision and power grips. It has been recognized that most manual grips can be divided in precision and power grips on the basis of functional, phylogenetic and developmental considerations [23]–[24]. In the power grip, a relatively large object is pressed between all fingers and the palm of the hand whereas the precision grip has developed in primates for the manipulation of small objects with the tip of the thumb and the index finger. It is important to notice that the double grasp neurons [15] discussed above are selective for the type of hand grip. The researchers distinguished three main groups of neurons in area F5: “Precision grip neurons”, “Finger prehension neurons”, “Whole hand prehension neurons”. In human, the double grasp processing of precision grasping in particular has been linked to preparation and execution of corresponding mouth movements [20]. For example, Higginbotham, Isaak and Domingue [25] observed an increase in electromyographic responses of the orbicularis oris muscles of the human participant during execution of precision grasping. Previously, in macaque, activation of these lip muscles has been associated with grasping a piece of food [26] and in human participants they are linked to the articulation of bilabial stops such as /p/ [27]. Furthermore, Waters and Fouts [28] proposed that similar double grasp activation can be observed in aimless sympathetic mouth movements during grasping. They reported that captive chimpanzees increasingly produced mouth movements such as protrusion and compression of the lips and tongue during fine manual manipulation particularly when the manipulation required the precision grip. Similar mouth movements that accompany the chimpanzees' precision grasping (e.g., lip protrusion) belong to the macaque communicative behavioural repertoire [29]–[31]. This suggests that in monkey the same mouth grasp gestures that are linked to corresponding hand grasp gestures for ingestive purposes are also employed for communicative purposes.

The double grasp preparation view is in line with the mouth-hand mimicry theory that was initially proposed by Wallace [32] and later elaborated by several other researchers [33]–[34] [5], according to which, there may be a natural tendency to mimic with the articulators what the hands are doing. This tendency may have led to utilization of articulatory gestures that bear at least crude relation to the hand gestures that they accompany. In other words, some articulatory gestures of speech may be initially built on representations of frequently observed hand actions such as precision and power grasping. In line with this view, Ramachandran and Hubbard [35] have proposed that “*…the oral gestures for ‘little’ or ‘diminutive’ or ‘teeny weeny’ synkinetically mimic the small pincer gesture…the flexion of the fingers and palmar crease in ‘come hither’ is mimicked by the manner in which the tongue goes back progressively on the palate*” (page, 21). In other words, they recognized that precision- and power-like hand actions (notice that in the power grip the fingers are brought towards a palmar crease) might be mimicked in certain articulatory gestures. According to this logic, if the double grasp neurons have been adapted for articulatory purposes at some point of speech evolution, it is likely that even the current repertoire of articulatory gestures would include gestures that are linked with the precision grasp and others that are linked with the power grasp.

Furthermore, this mouth-hand mimicry theory is in line with neurophysiological evidence showing that in monkey the same premotor circuits that are involved in preparation of hand movements are also involved in observing and imitating the model's hand movements [36]–[37] [26]. These so-called mirror neurons were recorded in the same F5 area as the double grasp neurons. Importantly, Ferrari et al. [31] showed that in addition to containing hand mirror neurons, the F5 also includes mouth mirror neurons that are associated with ingestive as well as communicative mouth gestures. In addition, some of these neurons have been observed to be activated by observing hand and mouth movements as far as they had the same goal [26]. Some of these neurons code actions in broadly congruent manner (e.g., observing and executing goal-directed grasps) whereas some of them code actions in strictly congruent manner (e.g., observing and executing goal-directed precision grasps) [26]. In addition, behavioural [38]–[39], brain imaging [40], transcranial magnetic stimulation [41], and single cell recording [42] studies have shown that a similar mirror neuron system can be also observed in humans. Hence, it is possible that a natural tendency to mimic with the articulators what the hands are doing – the observation that is proposed by the mouth-hand mimicry theory – is at least partially based on operations of these mirror neuron circuits. For example, it is plausible to assume according to the mirror circuit model that observing precision grasping might invite mimicking this hand action with the mouth, and this tendency might have added the precision grip related mouth gesture into the repertoire of articulatory gestures.

### The present study: the articulation-grip correspondence explored

The current study utilizes a behavioural compatibility paradigm in order to explore the hypothesis that the repertoire of articulatory gestures includes gestures that are associated with the precision grip and gestures that are associated with the power grip. The study employs a modification of the visuo-motor priming paradigm originally developed by Tucker and Ellis [43]–[46]. They investigated how the size of a perceived object influences manual responses executed with the precision or power grip devices. Their participants held both response devices in their dominant hand. Participants' task was to respond as fast as possible either with the precision or power grip device according to a certain property of the object (e.g., natural or man-made) that was presented on the computer monitor. They found that the execution of precision grip responses was facilitated when the size of the object was compatible with the precision grasping (e.g., strawberry) and the execution of power grip responses was facilitated when the size of the object was compatible with the power grasping (e.g., hammer). This finding suggested that an object's size automatically activates the grasp program that is compatible with the size of the object.

In the current study we were interested in whether articulation of consonant-vowel (CV) syllables would systematically influence response times and accuracy of precision or power grips performed simultaneously with the articulation. We expected that the congruency between the articulation and the grip type would influence the preparatory processes of grip selection in similar way as the size of the observed object influences grip selection processes in the study by Tucker and Ellis [43]. The influence of syllable pronunciation on executing different grip types has not been investigated before. Therefore, the selection of the syllables was based on the potential interplay between the precision and power grasping and the articulatory gestures in which the articulators are shaped in a similar way as these grip types. The syllables that were selected for the first experiment were [ti], [kα], [pu] and [mα]. We predicted that the syllables [ti] and [pu] would be more associated with the precision grip than with the power grip and the syllables [kα] and [mα] would be more associated with the power grip than with the precision grip for the following reasons. Firstly, we reasoned that the close vowel [i] would be more associated with the precision grip than with the power grip and the open vowel [α] would be more associated with the power grip than with the precision grip in the same way as they have been previously associated with small and large opening of finger aperture, respectively [22]. The vowel [i] is a close front vowel, which is formed with the tip of the tongue producing the constriction, resulting in a relatively small aperture in the vocal tract. The vowel [α] is an open back vowel, where the constriction is produced by the back of the tongue, resulting in a relatively large aperture in the vocal tract. Secondly, we hypothesized that the voiceless stop consonant [t] would be more associated with the precision grip than with the power grips as it is produced by bringing the tip of the tongue into contact with the alveolar ridge and the teeth. We speculated that this could be some sort of articulatory precision gesture, which is to some extent alike with grasp movement executed with the tips of the thumb and the index finger. A logical opposing pair for [t] is the voiceless stop [k], which is produced by moving the back of the tongue against the velum. Thus, we constructed a CV syllable using the consonant [t] together with the vowel [i], and an opposing syllable with consonant [k] together with the vowel [α]. There is a double opposition between apical and dorsal tongue gestures for the consonants as well as close versus open vocalic gestures, both of which involve the tongue and the jaw. The alveolar or dental closure of [t] mainly involves the apical area of the tongue, whereas the velar closure of [k] involves the whole tongue including its root. Thus, the production of [ti] can be compared to precision grasping with the tips of the index finger and the thumb, as opposed to producing [kα], which can be compared to using the whole hand as in the power grip.

The syllables [ti] and [kα] are largely associated with tongue and jaw movements and therefore we refer to them as ‘tongue as a primary articulator’ (TPA) syllables. We were also interested in exploring whether the potential interaction between articulation and grip type is linked to lip shape. Previous research has linked the precision grip to the activation of the orbicularis oris muscles of the lips [25]. Therefore, we selected syllables [pu] and [mα] as the second pair and refer to them as ‘lips as a primary articulator’ (LPA) syllables. The orbicularis oris muscles are dominantly involved in articulatory gestures that require lip protrusion. Articulating [pu] requires a mouth shape that we speculated to be precision-like: When pronouncing [pu], the lips form a small protruded, and round shape already during the consonant. In contrast, we reasoned that the syllable [mα] would be a lip-related counterpart for the syllable [kα] because when pronouncing [mα] lips form a non-protruded and wide shape. Thus, we predicted that articulation of the syllable [pu] would be more associated with the precision grip than with the power grip.

## Experiment 1

In Experiment 1, participants were required to pronounce the syllable that was presented on the computer monitor and simultaneously execute either a precision or a power grip response according to the color in which the syllable was written. The TPA- and LPA syllables were presented in different blocks. We were interested in observing whether grasp responses would be performed faster and more accurately in conditions in which the simultaneously articulated syllable and the grasp type were presumably congruent. We anticipated that the precision grip would be congruent with the syllables [ti] and [pu] whereas the power grip would be congruent with the syllables [kα] and [mα].

### Methods

#### Participants

Twelve naïve volunteers (one male), 22–50 years of age (mean age, 28.5 years), participated in the experiment. All participants were native speakers of Finnish and had normal or corrected- to-normal vision. One participant was left-handed. We obtained written informed consent from all participants in the present study. The study was approved by the Ethical Committee of the Institute of Behavioural Sciences at the University of Helsinki.

#### Apparatus, stimuli and procedure

**Figure 1:**
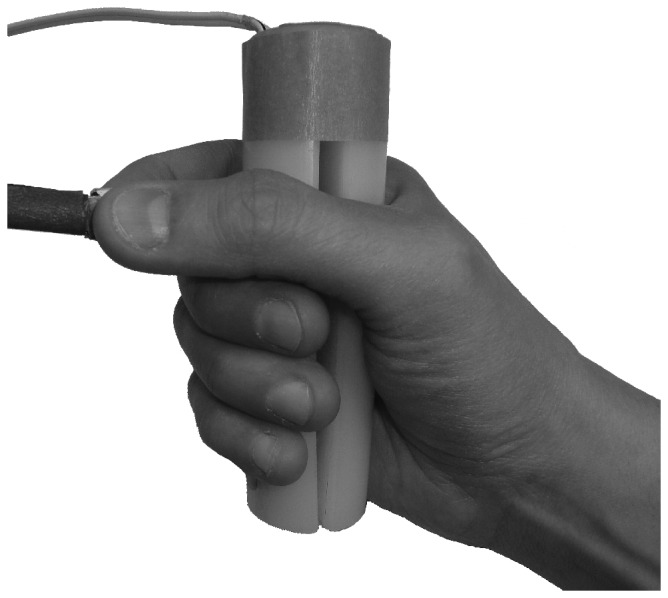
The response device used to record precision and power grip responses.

Each participant sat in a dimly lit room with his or her head 70 cm in front of a 19-in. CRT monitor. There were two response devices (Figure 1), each equipped with an inlaid micro-switch: the precision grip device (1×1×0.7 cm) and the power grip device (11 cm long, 3.2 cm diameter). As the switches were depressed in each device, there was noticeable tactile feedback. The devices were placed on a table in front of the participant.

We wanted to use a design that would most likely reveal any potential articulation-grip associations. So we chose a design in which TPA and LPA syllables were presented in separate blocks, and both blocks consisted of only one syllable that was anticipated to be related to the precision grip and one syllable that was anticipated to be related to the power grip. The stimuli consisted of orthographic syllables TI, KA, PU and MA that were written in the Arial font (bold; font size: 72). In one block (TPA), the stimuli TI and KA were presented in randomized order whereas in the other block (LPA) the stimuli PU and MA were presented in randomized order. There was a short break between the blocks. The order of the blocks was counterbalanced between the participants. Each stimulus was displayed 34 times. In total, the experiment consisted of 272 trials [34×4 (syllable) ×2 (grip type)].

A blank screen was displayed for 2000 ms at the beginning of each trial. Then the stimulus was presented at the screen centre for 400 ms in light grey color. Next the stimulus changed to either blue or green. Participants were holding both grip devices in their dominant hand. Their task was to respond as fast as possible according to the color of the stimulus. Half of the participants responded to the green with the precision grip and other half responded to the blue with the precision grip. The precision grip device was marked with a green tape and the power grip device with a blue tape for the precision-green group. Similarly, the precision grip device was marked with a blue tape and the power grip device with a green tape for the precision-blue group. The participants were instructed to respond with the green device to the green color and with the blue device to the blue color immediately when the color was perceived. The stimuli remained in view for 2000 ms or until a response was made. In addition, the participants were instructed to pronounce the presented syllable as fast as possible when it changed into color. It was emphasized that the syllable should be uttered in natural talking voice at the same time with the grasp response. Erroneous manual responses were immediately followed by a short “beep” tone.

The experiment began with practice trials. Each participant was given as much practice as it took to perform the task fluently. The experiment was not started before the participant was able to perform simultaneously accurate and fast manual and vocal responses. On average it took two minutes to develop sufficient level of skill in the task. Moreover, the experimenter was observing the task performance during the actual experiment and she immediately notified the participant if she noticed that the participant did not carry out the task accurately (i.e., the syllable production and the grasp response did not occur at the same time). The experimenter had to notify two participants during Experiment 1.

### Results

**Figure 2:**
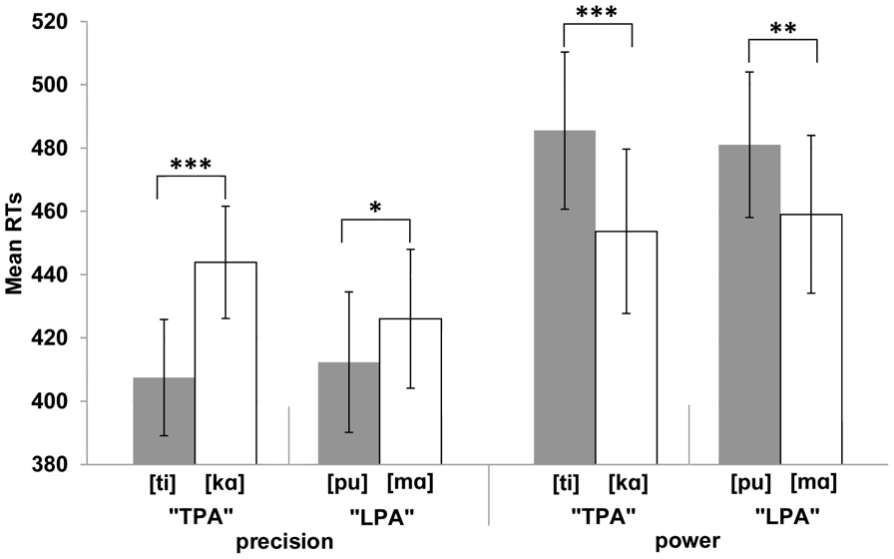
The mean reaction times (RTs) for **Experiment 1** **as a function of the syllable and the grip type.** The figure demonstrates that responses are performed faster when the syllable is congruent (e.g., [ti] – precision grip) rather than incongruent (e.g., [ti] – power grip) with the grip type. The lines between the syllable pairs indicate which syllables were presented in the same block. That is, the ‘tongue-as-primary-articulator’- (“TPA”: [ti] and [kα]) and ‘lips-as-primary-articulators’ (“LPA”: [pu] and [mα]) syllables were presented in different blocks. Bars refer to standard error of the mean. Asterisks indicate significance in the ANOVA (***p<.001; **p<.01; *p<.05).

In total, 6.8% of the raw data was discarded from the RT analysis including 2.6% of trials containing errors and 4.2% of trials in which the RTs were more than two standard deviations from a participant's overall mean. One participant did not make any errors. Condition means for the remaining data were subjected to a repeated-measures ANOVA with the within-participants factors of Syllable ([ti], [kα], [pu], [mα]) and Response (precision, power). This analysis revealed a significant main effect of Response, *F*(1,11) = 24.50, *MSE* = 53883.84, *p*<.001, η_p_^2^ = .690. Precision grip responses (M = 422 ms) were made faster than power grip responses (M = 470 ms). A similar main effect of response has been reported previously in studies that have measured precision and power grip responses [47]. Relatively fast precision grip responses are likely to reflect the fact that responding with the power grip device requires slightly more effort. That is, the difference is likely to be related to the mechanical aspects of the response devices rather than the neural resources related to planning precision and power grip responses. More importantly, the interaction between Syllable and Response was significant, *F*(3,33) = 25.21, *MSE* = 6033.67, *p*<.001, η_p_^2^ = .696. Precision grip responses were made faster when the syllables were [ti] (M = 407 ms) or [pu] (M = 412 ms) rather than [kα] (M = 444 ms) or [mα] (M = 426 ms). In contrast, power grip responses were made faster when the syllables were [kα] (M = 454 ms) or [mα] (M = 459 ms) rather than [ti] (M = 486 ms) or [pu] (M = 481 ms). This interaction is presented in Figure 2.

The interaction between Syllable and Response was also significant when the two blocks were analysed separately [TPA: *F*(1,11) = 54.97, *MSE* = 13985.08, *p*<.001, η_p_^2^ = .833; LPA: *F*(1,11) = 25.44, *MSE* = 3828.30, *p*<.001, η_p_^2^ = .698]. To examine this interaction more closely we carried out separate analysis of the simple main effects for each syllable pair within each block at each response type. The effect was significant for both syllable pairs in both response conditions [precision grip: TPA (*p*<.001) & LPA (*p* = .047); power grip: TPA (*p*<.001) & LPA (*p*<.01)]. We were also interested whether the strength of the two-way interaction that was observed in both blocks would differ between the blocks. Hence, we carried out an analysis for three-way interaction including the factors of Block (TPA, LPA), Anticipated Grip-relation of the Syllable (in the TPA block: precision [ti] and power [kα]; in the LPA block: precision [pu] and power [mα]) and Response (precision, power). This interaction was significant, *F*(1,11) = 6.36, *MSE* = 1589.65, *p = *.028, η_p_^2^ = .366. Given that the effect is smaller (see Figure 2) in the LPA block than in the TPA block, it appears that the correspondence effect between the articulatory gesture and the grip type is stronger for the TPA syllables than with the LPA syllables.

An analysis of percentage error rates revealed the pattern of results that was parallel to the results of the reaction time analysis. The error rates revealed a significant interaction between syllable and response, *F*(3,30) = 7.64, *MSE* = 71.66, *p* = .001, η_p_^2^ = .433. The participants made fewer errors with the precision grip when the syllables were [ti] (M = 0.5%) or [pu] (M = 0.3%) rather than [kα] (M = 3.2%) or [mα] (M = 1.6%). In contrast, the participants made fewer errors with the power grip when the syllables were [kα] (M = 1.3%) or [mα] (M = 2.1%) rather than [ti] (M = 5.9%) or [pu] (M = 5.6%).

### Discussion

The results of Experiment 1 demonstrated that articulation of certain syllables influences systematically simultaneously performed precision and power grip responses. Exactly as predicted, precision grip responses were made faster and more accurately when the pronounced syllables were [ti] and [pu] rather than [kα] and [mα]. Conversely, power grip responses were made faster and more accurately when the pronounced syllables were [kα] and [mα] rather than [ti] and [pu]. Furthermore, the results showed that even though the interaction between the syllables and grip types was significant in both blocks (TPA and LPA), the interaction was significantly stronger in the TPA block. In other words, it appears that the association between the articulatory gesture and the corresponding grip type is stronger in relation to TPA syllables ([ti] and [kα]) than LPA syllables ([pu] and [mα]).

Experiment 2 aimed to further investigate this finding by exploring which components in the syllables used in Experiment 1 had the strongest impact on precision and power grip responses. The syllables in Experiment 2 were constructed so that the consonants and vowels used in Experiment 1 were coupled with speech sounds that were reasoned to be relatively grip-neutral: vowel [e] and consonant [h]. Since we proposed that close front vowels would be associated with the precision grip whereas open back vowels would be associated with the power grip, the vowel [e] is in between these in terms of tongue height and, thus, should be relatively neutral regarding grip type. Consonant-vowel syllables consisting of [t], [k], [p] or [m] together with [e] were thus used to explore the effect of different consonants on grip responses. According to a similar logic, the fricative consonant [h] would be expected to be neutral with respect to grip types; [h] is produced by adjusting the vocal folds in a fashion that produces friction noise at the glottis as well as against the epiglottis and the vestibular folds. Neither the tongue nor the lips are thus directly involved in producing the sound and the laryngeal gesture adjusting the glottal opening does not resemble a grip in any conceivable manner [48]. Consonant [h] was thus used together with [i], [α] or [u] to explore the effect of different vowels in order to preserve the consonant-vowel syllable structure instead of just using the vowels alone. Therefore, the syllables that were used in Experiment 2 were [te], [ke], [pe], [me], [hi], [hα] and [hu].

## Experiment 2

### Methods

#### Participants

Fourteen naïve volunteers (one male), 22–58 years of age (mean age, 33 years), participated in the experiment. All participants were native speakers of Finnish, right-handed and had normal or corrected-to-normal vision. We obtained written informed consent from all participants in the present study. The study was approved by the Ethical Committee of the Institute of Behavioural Sciences at the University of Helsinki.

#### Apparatus, stimuli and procedure

The apparatus, stimuli and procedure were the same as those used in Experiment 1 with the following exceptions. The stimuli of Experiment 2 consisted of four blocks of syllable pairs. That is, the syllables were paired according to whether the articulation of the consonant was more associated with the tongue (“TPA”: [te]-[ke]) or lip (“LPA”: [pe]-[me]), or when different vowels were used together with the fricative consonant [h], and the vowel was close or open (“fricative/openness”: [hi]-[hα]), or rounded or unrounded (“fricative/roundedness”: [hu]-[hα]). The stimuli were again written in the Arial font (bold; font size: 72). The order of two syllables was randomized within the blocks whereas the block order was counterbalanced between the participants. There was a short break between the blocks. Each stimulus was displayed 30 times in each condition. In total, the experiment consisted of 480 trials [30×8 (syllable) ×2 (grip type)]. The experimenter had to notify three participants during Experiment 2 to focus on the synchrony between the pronunciation and the manual responses.

### Results

**Figure 3:**
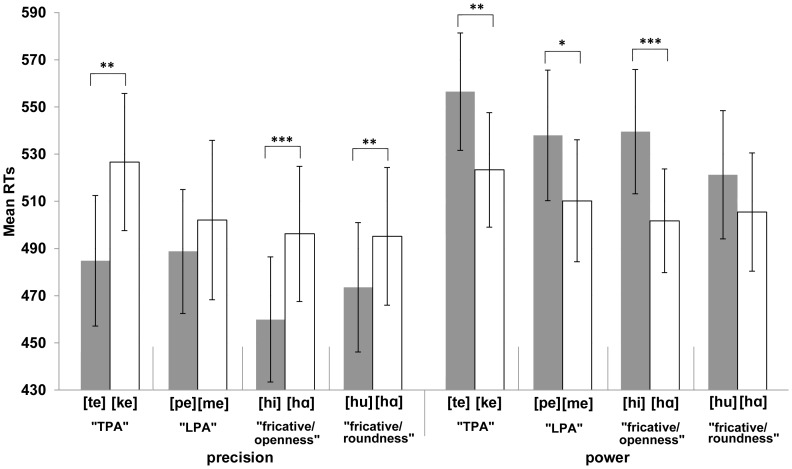
The mean reaction times (RTs) for **Experiment 2** **as a function of the syllable and the response type.** The figure demonstrates that responses are performed faster when the syllable is congruent (e.g., [te] – precision grip) rather than incongruent (e.g., [te] – power grip) with the grip type. The lines between the syllable pairs indicate which syllables were presented in the same block. That is, the ‘tongue-as-primary-articulator’- (“TPA”: [te] and [ke]) and ‘lips-as-primary-articulators’ (“LPA”: [pe] and [me]) syllables were presented in different blocks. In addition, the syllables that were used in the study in order to investigate the connections between the openness (“fricative/openness”: [hi] and [hα]) and roundness (“fricative/roundness”: [hu] and [hα]) of the vowels and the precision and power grip responses were also presented in their own blocks. Bars refer to standard error of the mean. Asterisks indicate significance in the ANOVA (***p<.001; **p<.01; *p<.05).

In total, 6.2% of the raw data was discarded from the RT analysis including 2.3% of trials containing errors and 3.9% of trials in which the RTs were more than two standard deviations from a participant's overall mean. One participant did not make any errors. Condition means for the remaining data were subjected to a repeated measures ANOVA with the within-participants factors of Syllable([te]-[ke], [pe]-[me], [hi]-[hα], [hu]-[hα]) and Response (precision, power). This analysis of reaction times revealed a significant main effect of Response [*F*(1,13) = 33.27, *MSE* = 63183.82, *p*<.001, η_p_^2^ = .719] and Syllable, *F*(7,91) = 2.30, *MSE* = 3200.57, *p* = .033, η_p_^2^ = .150. Similarly to the results of Experiment 1, the main effect of Response showed that precision grip responses (M = 491 ms) were made faster than power grip responses (M = 524 ms). Most importantly, the interaction between Syllable and Response was again significant, *F*(7,91) = 9.55, *MSE* = 7356.86, *p<*.001, η_p_^2^ = .424. In addition, the interaction between Syllable and Response was also significant when the four blocks were analysed separately. The pattern of the interaction was as predicted (Figure 3). In the TPA-block, precision grip responses were made faster when the syllable was [te] (M = 485 ms) rather than [ke] (M = 527 ms) and power grip responses were made faster when it was [ke] (M = 523 ms) rather than [te] (M = 556 ms), *F*(1,13) = 24.30, *MSE* = 19685.78, *p*<.001, η_p_^2^ = .651. In the LPA-block, precision grip responses were made faster when the syllable was [pe] (M = 488 ms) rather than [me] (M = 502 ms) and power grip responses were made faster when it was [me] (M = 510 ms) rather than [pe] (M = 538 ms), *F*(1,13) = 5.21, *MSE* = 5900.93, *p* = .040, η_p_^2^ = .286. In the block [hi]-[hα], precision grip responses were made faster when the syllable was [hi] (M = 460 ms) rather than [hα] (M = 496 ms) and power grip responses were made faster when it was [hα] (M = 502 ms) rather than [hi] (M = 540 ms), *F*(1,13) = 33.39, *MSE* = 19219.43, *p*<.001, η_p_^2^ = .720. In the block [hu]-[hα], precision grip responses were made faster when the syllable was [hu] (M = 474 ms) rather than [hα] (M = 495 ms) and power grip responses were made faster when it was [hα] (M = 505 ms) rather than [hu] (M = 521 ms), *F*(1,13) = 12.09, *MSE* = 4914.33, *p* = .004, η_p_^2^ = .482.

To examine this interaction more closely we carried out separate analyses of the simple main effects for each syllable pair within each block for each response type. In the precision grip response condition, the effect was significant for all syllable pairs except for [pe]-[me] ([te]-[ke]: *p* = .002; [pe]-[me]: *p* = .280; [hi]-[hα]: *p*<.001; [hu]-[hα]: *p* = .002). In the power grip response condition, the effect was significant for all syllable pairs except for [hu]-[hα] ([te]-[ke]: *p* = .003; [pe]-[me]: *p* = .016; [hi]-[hα]: *p* = .001; [hu]-[hα]: p = .092). We were also interested whether the strength of the two-way interaction that was observed in separate blocks would differ between the blocks. Hence, we carried out an analysis for three-way interaction including the factors of Block ([te-ke], [pe-me], [hi-hα], [hu-hα]), Anticipated Grip-relation of the Syllable (in the TPA block: precision [te] and power [ke]; in the LPA block: precision [pe] and power [me]; in the block [hi]-[hα]: precision [hi] and power [hα]; in the block [hu]-[hα]: precision [hu] and power [hα]) and Response (Precision, Power). This analysis revealed a significant interaction between the blocks [hi]-[hα] & [hu]-[hα] (*p* = .008), [te]-[ke] & [hu]-[hα] (*p* = .019) and [pe]-[me] & [hi]-[hα] (*p* = .015). The interaction was not significant between the blocks [te]-[ke] & [pe]-[me] (*p* = .150), [te]-[ke] & [hi]-[hα] (*p* = .964) and [pe]-[me] & [hu]-[hα] (*p* = .793). These interactions most likely reflect the fact, which can be observed in Figure 3, that the syllables [te]-[ke] and [hi]-[hα] provide the strong matches to the precision and power grip whereas the syllables [pe]-[me] and [hu]-[hα] provide the weaker matches to these grip types. This interpretation of the results is in line with the results of Experiment 1 in which the syllables [pu] and [mα] provided weaker matches to the grip types than the syllables [ti] and [kα]. However, one should be cautious to draw any stronger conclusions from these three-way interactions.

An analysis of percentage error rates showed a significant main effect of response type, *F*(1,12) = 7.24, *MSE* = 81.25, *p* = .020, η_p_^2^ = .376. Participants made fewer errors with the precision grip (M = 1.6%) than with the power grip (M = 2.9%). However, the interaction between syllable and response was not significant.

### Discussion

The results of Experiment 2 replicated the results of Experiment 1. Pronunciation of syllables again influenced precision and power grip performance and the interactions were as predicted. From the syllable pairs [te]-[ke], [pe]-[me], [hi]-[hα] and [hu]-[hα], the syllables [te], [pe], [hi] and [hu] were associated with relatively fast precision grip responses whereas the syllables [ke], [me] and [hα] were associated with relatively fast power grip responses. However, these interactions were not observed in errors like they were observed in Experiment 1. Taking into account the small overall percentage of errors (2.2%), not too much emphasis should be given to the absence of the effects in errors.

Similarly to the results of Experiment 1 in which the syllables [ti] and [kα] were linked to the clearest articulation-grip correspondence effect, the strongest associations were observed between the precision grip and the syllables [te] and [hi] as well as between the power grip and the syllables [ke] and [hα]. Thus, taking also into account the findings of Experiment 1, it appears that close vowels and consonants involving the tongue tip (apical speech sounds) are more associated with the precision grip than power grip whereas open vowels and velar consonants (dorsal speech sounds) are more associated with power grip than precision grip.

## General Discussion

The current study tested the hypothesis according to which some articulatory gestures are associated with the precision grip and some with the power grip. Both experiments showed a strong association between the grip type and the corresponding articulatory gesture. This occurred even though the grip responses were performed according to color cues so that the task did not require matching the articulated syllable with the grip type, suggesting that the correspondence operates in a relatively involuntary and automatic fashion. The clearest articulation-grip correspondence effects were associated with the following conditions: Those articulatory gestures in which the tip of the tongue is brought into contact with the alveolar ridge and the teeth or in which the aperture of the vocal tract remains relatively small ([ti], [te] and [hi]) were associated with relatively fast precision grip responses. In contrast, those articulatory gestures that are produced by moving the back of the tongue against the velum or in which the aperture of the vocal tract remains relatively large ([kα], [ke] and [hα]) were associated with relatively fast power grip responses. These results suggest that particularly the tongue shape and mouth opening for articulation is closely related to the precision and power grip.

In Experiment 1, a somewhat smaller but nevertheless significant articulation-grip correspondence effect was observed with the pronunciation of syllables in which the lips are the primary articulators. Pronunciation of the syllable [pu] was associated with relatively fast precision grip responses whereas pronunciation of the syllable [mα] was associated with relatively fast power grip responses. This finding is in line with the previous evidence that has linked lip protrusion movements [28] and lip muscles that are in dominant role in executing these types of lip movements [25] to precision grasping. Furthermore, the results of Experiment 2 were in line with the view according to which the precision grip is associated with speech sounds whose pronunciation requires a mouth shape in which lips are pushed forwards forming a small opening ([u]). The syllable [hu] was associated with significantly faster precision grip responses than the syllable [hα]. In addition, the lip-related syllable [me] that does not require strong lip protrusion was linked to faster power grip responses than the syllable [pe] in Experiment 2.

### A proposal for the neural mechanisms underlying the articulation-grip correspondence effect

We prefer to explain our results according to a modified version of the mouth-hand mimicry theory [5] [32]–[34], while also emphasizing the role of double grasp coding mechanisms [16]–[17]. We propose that the repertoire of articulatory gestures includes gestures that mimic frequently performed hand actions - the precision and power grip in particular. The natural tendency for mouth-hand mimicry and its utilization in speech is likely to be based on combined functions of the mirror neuron circuits and double grasp coding mechanisms that connect the corresponding hand and mouth actions. It is also possible that what we are observing in the articulation-grip phenomena is two effects that are partially based on different underlying neural mechanisms. In particular, those articulatory gestures that are mostly associated with tongue shape ([te] and [ke]) can be nicely explained by the mouth-hand mimicry processes. In contrast, double grasp processes that originally serve eating behavior might be more suitable explanation for those articulatory gestures that are mostly related to the openness of the mouth ([hi] and [hα]), given that the size of the object provides similar grasp requirements for the hand and the mouth when the object is put into the mouth. More research is needed to reveal whether the same mechanism is causing all articulation-grip phenomena observed in the present study or whether some of them are more based on mimicry processes and others are more based on double grasp coding processes.

Although our findings fit nicely with the hypotheses that can be drawn from the mouth-hand mimicry theory as well as the double grasp coding findings, one should be aware of that it is always extremely difficult to solidly identify the precise causes of this kind of behavioural congruency effects. Firstly, the effect reveals a systematic association between two motor processes rather than providing detailed information about excitatory and/or inhibitory components that are operating behind the effect. Moreover, in addition to the fact that researchers have only indirect evidence for the existence of the double grasp neurons in human, the double grasp neurons were originally associated with preparation processes of hand prehension that require computations on the shaping of the hand [15], rather than just a selection of the already shaped grip type as it was the case in the current study. However, double grasp neurons are involved in preparatory action operations and broadly represent manual-movement prototypes rather than organize precise hand kinematics. In fact, these neurons are also activated by visual stimuli if the size of the stimulus matches the type of the movement that the neuron codes. For example, a neuron that is involved in preparing the precision grip actions responds only to a visual stimulus whose size is congruent with the precision grip. This suggests that these neurons are involved in selecting the motor act that allows the individual to take possession of the object (e.g., selecting the type of grip). Therefore, it is plausible to assume that in the current study the selection of the already shaped grip type would be computed by the mechanisms that are similar to the double grasp preparation mechanisms observed in monkey.

On the other hand, it could be speculated that the articulation-grip correspondence phenomenon may reflect some more abstract cognitive operations than what has been suggested above. The effect might be based on operations that provide semantic size information in a generalized manner for different cognitive functions such as thinking, acting and language. For instance, the articulation-grip correspondence phenomenon might be parallel to the SNARC (Spatial-Numerical Association of Response Codes) phenomenon in which large numbers are automatically associated with the right side of space and small numbers with the left side of space [49]. A similar effect to the SNARC, which is even more relevant to the present articulation-grip phenomenon, has been reported by Moretto and Di Pellegrino [47] [50] showing that precision grip responses were performed faster when the participants were presented with numbers of small rather than large numerical value whereas power grip responses were performed faster when the participants were presented with numbers with large rather than small numerical value. This finding was proposed to demonstrate that semantic knowledge of magnitude information is represented sensory-motorically in the system that also represents action plans. Corresponding demonstrations have been observed in numerous semantic priming effects in which the semantic information about, for example, the direction or size of the word that is presented to the participant influences planning processes of manual action [21] [44] [51]–[52]. However, it should be reminded that in the current study, the articulated syllables did not include any obvious size/shape information that might have influenced the grip selection processes other than size/shape information related to the articulatory movements. Hence, we are prone to assume that the underlying neural mechanisms of the articulation-grip correspondence effect can be separated from the mechanisms associated with the above mentioned semantic sensory-motor priming effects even though they may also partially share overlapping mechanisms.

Out interpretation of the results also motivates to ask whether this articulation-grip phenomenon has played some role in the evolution of speech. It is possible that speech has started to evolve to its present form when the simple utterances in the proto-speech have started to utilize the already existing motor mechanisms of manual system including particularly the repertoire of grasp representations. However, we are cautious in taking any strong stance on these evolutionary questions. For example, the current results cannot resolve the question whether language has evolved directly as speech or whether manual communication has preceded the vocal communication. However, the results support the view that at least some articulatory gestures are grounded in manual grip types. During the evolution of speech, this connection might have increased the complexity of available utterances in speech.

### A proposal for the potential role of grasp mechanisms in the previously observed correspondence between sounds and visual properties of objects

The current findings can be linked to the so-called sound symbolism phenomena according to which spoken words have arisen from congruencies between sound and meaning [54]. This idea challenges the traditional view according to which phonetic units are mostly mapped arbitrarily to the objects they are referring to [54]–[55]. For instance, those words that refer to something small frequently consist of close vowels (e.g., little) whereas the words referring to something large frequently consist of open vowels (e.g., large). In line with this observation, [53] demonstrated that a nonsense word containing a close vowel (*mil*) is more likely to be associated with a small object and a word containing an open vowel (*mal*) is more likely to be associated with a large object. Recently, Peña, Mehler and Nespor [56] found that infants matched close vowels with small object size and open vowels with large object size. Parallel to this, probably the best-known demonstration of biased sound-shape mappings is Köhler's [57]–[58] finding that nonsense words such as *takete* and *maluma* are reliably matched to images of unfamiliar jagged and curved objects, respectively.

It has been suggested that the correspondence between sound and visual properties of objects is observed because the mechanisms that produce the speaker's lip and tongue movements are tightly connected to the perceptual system that represents visual properties of objects perhaps via sound representations associated with different phonetic gestures [35]. According to this view certain words such as ‘petite’, ‘little’ and ‘diminutive’ have developed to refer to small objects because the opening of the vocal tract is small when these words are pronounced. In addition to connecting the meaning of some words and the shape of the vocal apparatus, Ramachandran and Hubbard [35] also linked the phenomenon to manual gestures. For example, they recognized that the oral gestures for articulating certain adjectives such as ‘tiny’ might mimic the precision grip gesture. In addition, Gentilucci and Campione [22] proposed that their finding concerning the systematic interaction between pronunciation of open and close vowels and openness of the finger aperture may partially support the sound symbolism theory. Similarly, the results of the current study show that certain articulatory gestures are systematically associated with different grip types, in the same way as they are associated with different object shapes and sizes. The sound-size correspondence research has linked the close vowel [i] to small objects and the open vowel [α] to large objects [53]. In line with this, the present study linked the close vowel [i] to the precision grip and the open vowel [α] to the power grip. Furthermore, the precision grip may be more associated with object shapes with sharp limbs than the power grip because grasping this kind of objects requires cautious and precise prehension. If so, it could be speculated that the current data is similarly in line with the sound-shape correspondence evidence which has linked the stop consonant [t] to jagged shapes and the continuant consonant [m] to curved shapes [59]. In the same way, the present data linked the stop consonant [t] to the precision grip and the continuant consonant [m] to the power grip.

The interpretation of double grasp neurons linking them to certain articulatory gestures [16]–[17] implicitly supports the idea that the double grasp preparation processes can play a role in certain size- and shape-related sound symbolism phenomena. That is, because these neurons do not only reveal a tight interplay between hand grasp and mouth movements, but some of them also respond selectively to the size of the viewed objects linking visual object properties to the preparation of hand grasping as well as mouth movements. Hence, it could be speculated that viewing a small object might amplify articulatory gestures that are congruent with the size (e.g., little) when an individual produces referential utterances in relation to this object. Furthermore, it is important to highlight that selection of the grasp type is naturally almost entirely determined by the size or shape of the goal object whereas articulations *can be* selected arbitrarily in relation to the reference object. In addition, the research has demonstrated a strong connection between visual properties of objects (e.g., size) and the hand grasp preparation [15] [43] [60]–[61]. Thus, it is even possible that the manual grasp component of the double grasp coding system has played a dominant role in the development of certain size- and shape-related sound symbolism phenomena. That is, in some sound symbolism cases, the manual grasp component of the double grasp preparation system might have instructed the articulatory processes to match them with the hand grasp as well as the visual property of an object leading to sound symbolism phenomena. For example, seeing a small object amplifies the representation of the precision grip which in turn amplifies the representation of articulatory gesture [i] when producing a referential utterance in relation to the object. Therefore, we suggest that in addition to linking certain sound symbolism phenomena to the shape of the articulatory gestures, the manual grasp processes should be also considered as the central component in these phenomena.

## Conclusion

To sum up, the current study presents a novel articulation-grip correspondence effect showing systematic interactions between articulatory gestures and grip types. The clearest articulation-grip correspondence effects were associated the articulatory gestures in which the tip of the tongue is brought into contact with the alveolar ridge and the teeth or in which the aperture of the vocal tract remains relatively small ([ti], [te] and [hi]). These gestures were associated with relatively fast precision grip responses. In contrast, relatively fast power grip responses were associated with gestures that are produced by moving the back of the tongue against the velum or in which the aperture of the vocal tract remains relatively large ([kα], [ke] and [hα]). In addition, there was a less strong association between the precision grip and the articulatory gesture that requires lip protrusion movements ([pu]). These effects fit nicely with the hypotheses that can be drawn from the mouth-hand mimicry theory [5] [32]–[34] as well as the double grasp coding findings [16]–[17] [25] [28]. Finally, we propose that the present findings might clarify the sound symbolism theory by adding the hand grasp component to the mechanisms on which certain sound symbolism phenomena are grounded.
